# Diffuse Idiopathic Aneurism of the Anterior Tibial Artery

**Published:** 1881-08

**Authors:** G. Frank Lydston

**Affiliations:** Late House Surgeon Charity Hospital; Resident Surgeon, State Emigrant Refuge and Hospital, Ward’s Island, New York


### ARTICLE III.

### DIFFUSE IDIOPATHIC ANEURISM OF THE ANTERIOR TIBIAL ARTERY. TETANUS FOLLOWING AMPUTATION OF THIGH.

By G. FRANK LYDSTON, M.D., late House Surgeon Charity Hospital; Resident Surgeon, State Emigrant Refuge and Hospital, Ward's Island, New York.

The patient, T. R., a painter by occupation, and thirty years of age, entered Charity Hospital, September 4, 1880. He gave a history of having had malaria and rheumatism, and several attacks of gonorrhœa, but said that he had been otherwise healthy until four years since, when he contracted several sores upon his penis, which were termed "mixed sores," by his physician. In about eight weeks thereafter, he developed secondary syphilis, which was treated by a mercurial course with apparent success. Some time afterward however, he developed several tertiary ulcers upon his legs, for which he was treated in the hospital, and which healed readily in a few weeks. About eight weeks previous to last admission, several ulcers appeared upon the outer aspect of the left leg, just below the knee over the heads of the tibia and fibula. These were evidently syphilitic in character, and he was accordingly placed upon the "mixed treatment," with a large excess of the iodide of potassium. The benefit from this was only temporary, the ulcers soon beginning to extend, and the bone becoming involved. The odor from the ulcers soon became so offensive that it became necessary to transfer the case to the surgical pavilion. During December, six arterial hæmorrhages occurred from the central ulcer over the head of the tibia, which on several occasions were difficult to control. Necrosed bone was now plainly visible upon the heads of both tibia and fibula at the bottom of the ulcer. On December 10, a consultation was called. In the situation of the ulcer, there was now a large elastic tumor, and the finger being introduced into the ulcer, portions of blood-clot were detached, which seemed to be a portion of the tumor. Exploration decided that the tumor contained blood. During the examination, and while the limb was not under manipulation, severe arterial hæmorrhages occurred, rendering the application of a tourniquet necessary. The diagnosis of "diffuse aneurism" was made, and as the condition of the parts rendered deligation of the femoral unpromising, amputation of the thigh, in the lower third, was determined upon. On December 14 the operation was performed; Esmarch's "bloodless method," and the Lister spray and dressing being used. The amputation was made with lateral skin flaps, the femur being divided at about the middle of its lower third. The patient reacted from the anæsthetic very slowly, and the pulse being very feeble ℥ss of brandy was given. The subsequent course of the case was as follows:

December 15. Temperature 103° F.; pulse 128. Patient feeling comfortable.

December 16. Temperature 101°; pulse 100. Dressings removed and stump redressed under the carbolic spray. Stump looking well.

December 17. Temperature 101.4°; pulse 116. Patient feeling well.

December 18. Temperature 101.5°; pulse 110. Stump redressed and all stitches removed excepting two at upper angle of wound, on account of sloughing of the flaps to some extent.

December 19. Temperature 100.5°; pulse 112.

Decembor 20. Temperature 100°; pulse 104. Stump redressed and dressings ordered to be renewed three times daily. Stump looks fair, but considerable suppuration going on.

December 21. Temperature 100°; pulse 104. Patient quite comfortable.

December 22. Patient complained of a sense of depression, and expressed the fear "that something was going to happen to him," but he had no definite symptoms. Temperature 100°; pulse 100.

December 23. Patient complained of "tingling and twitching sensations in the stump, and of some stiffness and soreness of jaws. Countenance anxious and pinched. Temperature 100°; pulse 110. Was ordered sol. Magendie min. x, t. d.

December 24. Patient much worse. Trismus still more marked than on day previous. Some involuntary spasms of the extremities, notably of the affected thigh, have occurred since last visit. Countenance more pinched, and extremely sallow. Temperature 100°; pulse 112. General appearance suggests a certain degree of septicæmia.

December 25. Several slight convulsions of limbs occurred during the night of the 24th. Jaws cannot now be opened sufficiently for the introduction of food. Fluids are expelled when drunk, and return partially through nostrils, suggesting paresis of soft palate. Any attempt to force open the jaws caused a slight general convulsion without loss of consciousness, and of short duration. Patient very feeble, and evidently sinking rapidly. Rectal alimentation and stimulation were resorted to, but to no purpose. Chloral hydrate was also administered per enema. Temperature 104°; pulse 125. Death occurred on this date, 25th, at 11 P. M.

No severe muscular spasms occurred at any time, and there was no opisthotonos. The tetanoid symptoms were, I think, modified in great measure, by the septicæmic element which evidently existed. No autopsy was held, but an examination of the diseased limb was made shortly after its removal.

It was found that an ulceration and solution of continuity of the anterior tibial artery, about half an inch from its origin, had occurred. The eroded ends of the artery were found free, and connecting with a cavity about as large as a good sized orange, which was filled with disorganized blood-clot, occupying the inter-osseous space at the heads of the bones, the space between which was greatly enlarged from necrosis. This cavity had been formed by the pressure of its contained blood, the muscles having been displaced in its formation, and a pseudo sac formed by plastic deposit in the surrounding tissues. The degeneration of the artery was of considerable extent, there being some distance between the distal and proximal extremities in this situation, with portions of the degenerated arterial coats, present in the contents of the sac.

